# Infection by *Toxoplasma gondii* Induces Amoeboid-Like Migration of Dendritic Cells in a Three-Dimensional Collagen Matrix

**DOI:** 10.1371/journal.pone.0139104

**Published:** 2015-09-25

**Authors:** Sachie Kanatani, Per Uhlén, Antonio Barragan

**Affiliations:** 1 Department of Molecular Biosciences, The Wenner-Gren Institute, Stockholm University, Stockholm, Sweden; 2 Center for Infectious Medicine, Department of Medicine, Karolinska Institutet, Stockholm, Sweden; 3 Laboratory of Molecular Neurobiology, Department of Medical Biochemistry and Biophysics, Karolinska Institutet, Stockholm, Sweden; Centre National de la Recherche Scientifique, FRANCE

## Abstract

*Toxoplasma gondii*, an obligate intracellular parasite of humans and other warm-blooded vertebrates, invades a variety of cell types in the organism, including immune cells. Notably, dendritic cells (DCs) infected by *T*. *gondii* acquire a hypermigratory phenotype that potentiates parasite dissemination by a ‘Trojan horse’ type of mechanism in mice. Previous studies have demonstrated that, shortly after parasite invasion, infected DCs exhibit hypermotility in 2-dimensional confinements *in vitro* and enhanced transmigration in transwell systems. However, interstitial migration *in vivo* involves interactions with the extracellular matrix in a 3-dimensional (3D) space. We have developed a collagen matrix-based assay in a 96-well plate format that allows quantitative locomotion analyses of infected DCs in a 3D confinement over time. We report that active invasion of DCs by *T*. *gondii* tachyzoites induces enhanced migration of infected DCs in the collagen matrix. Parasites of genotype II induced superior DC migratory distances than type I parasites. Moreover, *Toxoplasma*-induced hypermigration of DCs was further potentiated in the presence of the CCR7 chemotactic cue CCL19. Blocking antibodies to integrins (CD11a, CD11b, CD18, CD29, CD49b) insignificantly affected migration of infected DCs in the 3D matrix, contrasting with their inhibitory effects on adhesion in 2D assays. Morphological analyses of infected DCs in the matrix were consistent with the acquisition of an amoeboid-like migratory phenotype. Altogether, the present data show that the *Toxoplasma*-induced hypermigratory phenotype in a 3D matrix is consistent with integrin-independent amoeboid DC migration with maintained responsiveness to chemotactic and chemokinetic cues. The data support the hypothesis that induction of amoeboid hypermigration and chemotaxis/chemokinesis in infected DCs potentiates the dissemination of *T*. *gondii*.

## Introduction

The obligate intracellular parasite *Toxoplasma gondii* causes infections in warm-blooded vertebrates and chronically infects a large portion of the global human population [1]. The dissemination of the parasite from the point of entry in the intestinal tract plays a determinant role in the pathogenesis of toxoplasmosis. Severe manifestations such as encephalitis occur in the central nervous system of immune-compromised individuals and ocular pathology such as retinochoroiditis manifests in otherwise healthy individuals. Congenital toxoplasmosis occurs by transmission to the fetus from the infected mother and can result in severe disabilities or death of the unborn child [2].

Previous studies have demonstrated that active invasion of dendritic cells (DCs) by *T*. *gondii* tachyzoites rapidly (within minutes) induces a hypermigratory phenotype in DCs [3]. This migratory activation is characterized by cytoskeletal rearrangements, dramatically enhanced cellular locomotion on 2D surfaces, termed hypermotility [4], and enhanced transmigratory activity *in vitro* [5]. In murine models of toxoplasmosis and neosporosis, the hypermigratory phenotype has been linked to enhanced dissemination and increased parasitic loads [6–8]. The initiation of the hypermigratory phenotype in DCs is related to the discharge of secretory organelles during parasite invasion and does not depend on *de novo* protein synthesis in the host cell [4]. It is mediated through non-canonical GABAergic signaling pathways, and is independent of MyD88-mediated TLR signaling and chemotaxis [3–5, 7].

DCs likely play a pivotal role during *T*. *gondii* infection as mediators of essential immune responses [9, 10] and as parasite carriers that facilitate the dissemination of the infection [5, 8, 11, 12]. As a fundamental component of the immune response, DCs sense, sample and process antigens in peripheral tissues for initiation of adaptive immune responses and pathogen clearance [13]. The mechanisms underlying DC maturation and migration are complex, and the molecular trafficking signals that govern DC migration are not fully understood [14]. One of the hallmarks of maturing DCs is the expression of the C-C chemokine receptor 7 (CCR7). Chemokinetic and chemotactic effects following binding of CCR7 to its ligands (CCL19 and CCL21) promote motility and guide the migrating cells across interstitial tissues to the secondary lymphoid organs where adaptive immune response is initiated [14, 15].

The switch from an immature state to a mature state requires major alterations in the actin cytoskeleton of DCs, thereby allowing the DCs to cross extracellular matrix when migrating from the periphery to the lymphatic circulation or from the blood into tissues [14]. Collagen is a major component of extracellular matrix. The integrin family of cell adhesion molecules chiefly mediates the cellular interactions with collagen. While DC migration on two-dimensional (2D) substrates shows dependency on integrin binding, DC migration in three-dimensional (3D) environments exhibits different characteristics [16]. The change in shape that accompanies rapid leukocyte migration has been termed “amoeboid” [17]. In contrast to other migration modes, amoeboid movement is particularly suited for rapid locomotion of leukocytes in cellular networks and tissues [18]. More recent work has shown that amoeboid motility of DCs occurs independently of integrin-mediated adhesion to specific substrates and of extracellular matrix degradation [18], and is required for efficient migration [19]. Consequently, interstitial migration of DCs was suggested to be autonomous from the molecular composition of the extracellular environment and chiefly dependent on the protrusive flow of the actin cytoskeleton [16, 20].

Because DCs have been attributed a shuttling function in the dissemination of *T*. *gondii*, we have functionally tested and characterized the migration of *Toxoplasma*-infected DCs in a 3D collagen matrix. We report that an amoeboid-like hypermigration is induced in *Toxoplasma*-infected DCs.

## Materials and Methods

### Ethics statement

The Regional Ethics Committee, Stockholm, Sweden, approved protocols involving human cells (application number 2006/116-31). All donors received written and oral information upon donation of blood at the Karolinska University Hospital Blood Center (http://www.karolinska.se/for-vardgivare/Karolinska-Universitetslaboratoriet/). Written consent was obtained for utilization of white blood cells for research purposes.

### Parasites and reagents

*T*. *gondii* lines used include GFP-expressing RH-LDMluc (type I, cloned from RH-GFPS65T) [21], GFP-expressing PTGluc (type II, cloned from ME49/PTG-GFPS65T) [21] and RFP-expressing PRU-RFP (type II) [22]. Tachyzoites were maintained by serial 2-day passaging in murine fibroblasts (L929, Sigma-Aldrich) cultured in Dulbecco's modified Eagle's medium (DMEM; Thermofisher scientific) with 10% fetal bovine serum (FBS; Sigma), gentamicin (20 μg/ml; Gibco), glutamine (2 mM; Gibco), and HEPES (0.01 M; Gibco), referred to as complete medium (CM).

Antibodies used include anti-human CD11a, CD11b, CD18, CD49b, mouse IgG1 κ Isotype, rat IgG2b κ Isotype (all BioLegend) and anti-human CD29 (R&D Systems). All antibodies were added at a concentration of 10 μg/ml in the assays.

### Primary DCs

To generate human monocyte-derived DCs, buffy coats from healthy blood donors were incubated with monocyte enrichment cocktail (RosetteSep^TM^, StemCell Technologies), followed by centrifugation on density gradient medium (Lymphoprep^TM^, StemCell Technologies). The in-between layer containing monocytes was transferred and washed twice with phosphate buffered saline (PBS). The residual red blood cells were removed by using red blood cell lysis buffer (15 mM NH_4_Cl, 1.4 mM NaHCO_3_, 0.03 mM EDTA, pH7.3). The cell population obtained was composed mainly of CD14^+^ (DakoCytomation) with < 1% CD3^+^/CD19^+^ cells (BD), as evaluated by flow cytometry (FACS-Calibur, BD). DCs were generated by culturing the purified population in DMEM supplemented with 75 ng/ml GM-CSF (PeproTech) and 30 ng/ml IL-4 (PeproTech) for 6 days. The medium was changed after 3 days in culture.

### 3D matrix migration assay

A collagen layer was prepared by mixing bovine collagen I (0.75 mg/ml; Gibco) in PBS and pH was adjusted to 7.0–7.5. Cold collagen solution was poured into a 96-well plate (80 μl/well) avoiding air bubble formation. Gel formation at 37°C for 1 h was allowed before start of the assays. DCs were challenged with freshly egressed *T*. *gondii* tachyzoites in CM for 4 h at multiplicity of infection (MOI) 3. The infection frequency of DCs was determined by epifluorescence microscopy (S2 Fig). The cell suspension (5×10^4^ cells) was applied to the collagen layer and incubated for 18 h at 37°C and 5% CO_2_. The medium was gently aspirated and the gels were fixed by addition of 100 μl of 4% paraformaldehyde in PBS. Gels were then washed once with PBS, stained with 57.2 nM DAPI in PBS and stored at 4°C.

To establish a chemotaxis gradient, 40 μl of collagen solution containing 3 mg/ml CCL19 (Peprotech) was casted into a 96-well plate, allowed to polymerize, and 40 μl of collagen solution was overlaid. The migration assay was performed for 24 h at 37°C and 5% CO_2_.

### Image acquisition and migration analysis

After fixation and staining, image stacks were generated (200 optical sections, 3.48 μm/section) using a 10X objective positioned under the center of the well, using a Zeiss LSM700 or LSM 780 laser scanning confocal microscope. For each well, the layer of cells at the top of the matrix that had not penetrated the matrix defined the starting location at the top of the gel (0 μm) of the migrating cells, e.g. the most distant point from the bottom of the well containing cells. Z-stack images were exported to Imaris x64 v. 8.1.1 sofware (Bitplane AG, Zurich, Switzerland) and 3D image projections (x: 848.53 μm, y: 848.53 μm, z: 692.52 μm) were created by Imaris Surface module. The position of cells in the 3D confinement was calculated by Imaris Spot module. For each cell, the migrated distance was defined the Euclidian distance from the top of the matrix (0 μm) to the identified x,y,z-position of the cell in the matrix. An average of 500 (range 400–800) cells were analyzed per experiment. For each experiment, 400–500 randomly selected cells (RAND function, Microsoft Excel) were used for calculating mean migrated distances.

For DCs challenged with *T*. *gondii*, a semi-automated algorithm was generated to analyze infected and non-infected cells using the Imaris Spots Colocalize module. First, the Euclidian center of phalloidin-stained cells (DC, RFP or GFP) was determined. An infected cell was defined as presence of signal (parasite, GFP/type I or RFP/type II) overlapping with signal (DC, RFP or GFP) within a radius of 20 μm from the center point of the cell. A non-infected cell was defined as absence of signal (parasite, GFP/type I or RFP/type II) within a radius of 20 μm from the center point of the cell. Visual confirmation was performed before final analysis.

### Immunocytochemistry and cell morphology scoring

Cold collagen I solution (0.75 mg/ml) was poured into a glass bottom dish (100 μl/dish) and was allowed to polymerize at 37°C for 1h. DCs were challenged with freshly egressed *T*. *gondii* in CM for 4 h at MOI 3. The cell suspension were applied to a glass bottom dish and incubated for 18 h at 37°C and 5% CO_2_. Fixation was performed with 4% paraformaldehyde in PBS and gels were permeabilized by using 1% Triton X-100 (Sigma) in PBS. Gels were then washed once with PBS, stained with Alexa Fluor 594-conjugated phalloidin (Lifetechnology), DAPI and stored at stored at 4°C. Image stacks were generated using a 63X objective (10 optical sections, 0.45 μm/section) or a 40X objective (5–10 optical sections, 0.93 μm/section) on a Zeiss LSM 780 laser scanning confocal microscope. Z-stack images were processed and were projected with maximum-intensity projection by Zen black software.

For cell morphology scoring, 20–30 randomly chosen fields of view were acquired from each preparation. 3D images in maximum intensity projection were created from z-stack images and 50 cells from each individual donor were graded as previously described [4] with some modifications. Briefly, the DCs were scored based on three morphological criteria (A-C):

- Cell shape—elongated (score 0) versus rounded (score 1).
- Membrane extensions—present (score 0) versus absent (score 1).
- Membrane veils—absent (score 0) versus present (score 1)

A final score resulted in the addition of all three (A-C) criteria and therefore could range from 0–3.

### Adhesion assay

96-well flat-bottom plates were coated with 1% denatured bovine serum albumin (BSA, Sigma) in PBS or collagen I (5μg/cm^2^; Gibco) for 1h at RT [23]. The wells were washed twice with DMEM (Thermofisher scientific) and the cell suspension (2x10^4^ cells) was allowed to attach at 37°C, 5% CO_2_ for 15 min. Unbound cells were removed by washing twice with DMEM, once with PBS. Attached cells were fixed with 4% paraformaldehyde, DAPI-stained, imaged using a Zeiss LSM700 microscope and analyzed with Image J software (NIH, MD, USA).

### Statistical analyses

Statistical analyses were performed using R Stats package version 3.0.2 (R Foundation for Statistical Computing, Vienna, Austria).

## Results

### Assessment of active DC migration in a 3D collagen matrix

**Fig 1:**
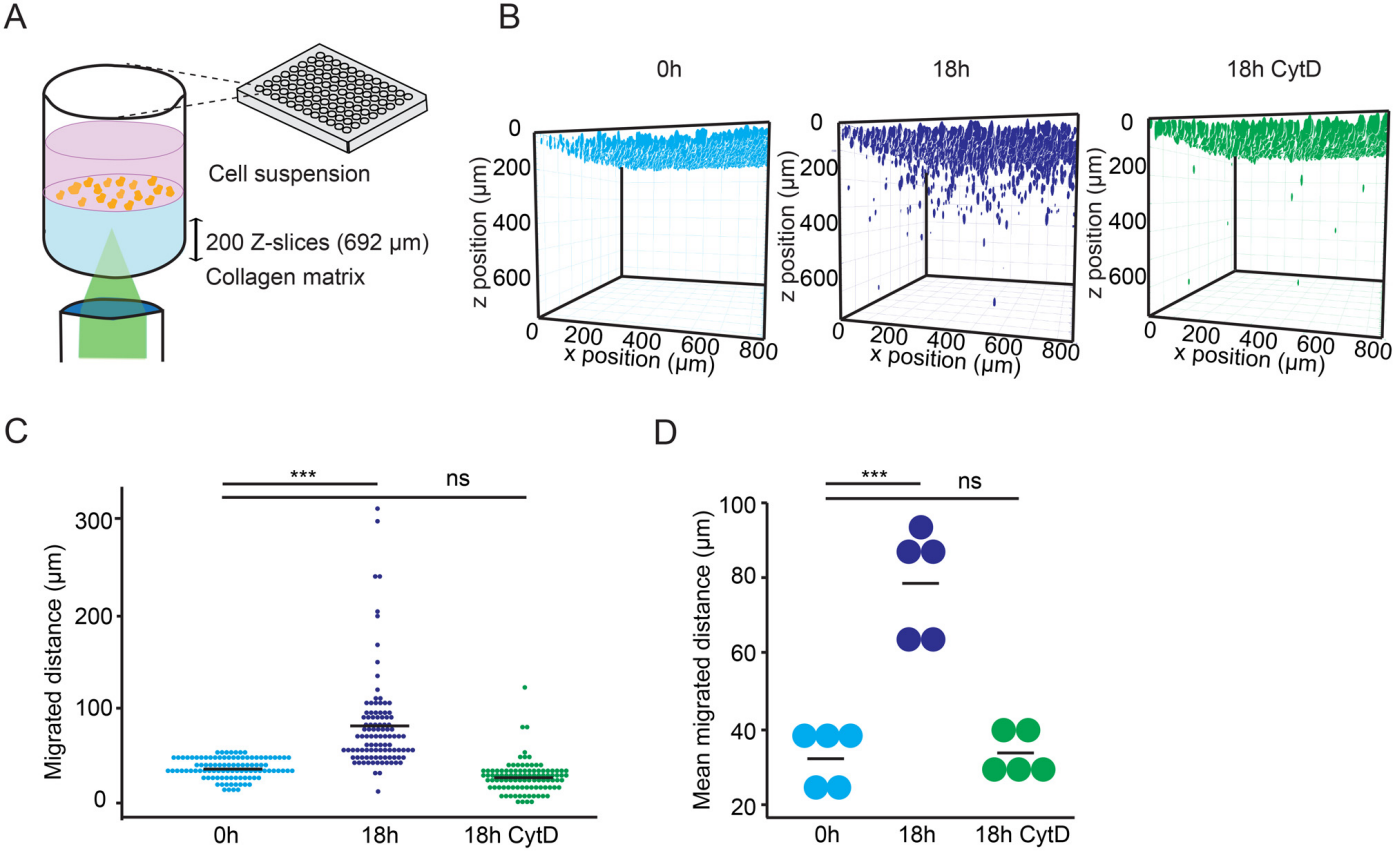
Experimental set-up for DC migration in a 3D collagen matrix. (**A**) Schematic representation of the assay set-up with collagen matrix as indicated under Materials and Methods. DCs were maintained in complete medium (CM) and allowed to sediment on the top of the collagen matrix layer in 96-well plates. After 18 h incubation, the localization of DAPI-labeled DCs in the collagen gel was analyzed by confocal microscopy in 200 z-optical sections as indicated. (**B**) Plots represent, for each condition, the 3D reconstruction assembly of z-stacks as indicated under Materials and Methods. Colored structures indicate the localization of individual DCs (DAPI) at indicated time points ± cytochalasin D (CytD). (**C**) Dot plots represent the distribution of migrated distances for the different conditions. For each condition, 100 single cells were randomly selected and analyzed from one representative donor. Bar indicates mean migrated distance. Asterisks indicate significant differences (***: P < 0.001; non-significant (ns): P > 0.05 Kruskal-Wallis test, Dunnett´s test). (**D**) Mean migrated distances by DCs under same conditions as in B and C. Data represent compiled analysis of 500 randomly chosen cells per donor from 5 different donors. Bars indicate mean migrated distances (***: P < 0.001, ns: P > 0.05; Two-way ANOVA, Tukey´s HSD test).

To assess the migratory properties of human monocyte-derived DCs in a 3D collagen matrix set-up (Fig 1A), the DCs were applied to the top of a collagen matrix and their position within the matrix space was automatically determined at indicated time-points (S1 Fig). Penetration of the matrix by DCs was observed by 18 h compared with time point 0 h, and penetration was abrogated in the presence of cytochalasin D, an inhibitor of actin polymerization (Fig 1B). Significant differences in the mean migrated distances were observed over time (time point 0 h vs. 18 h), while cytochalasin D-treatment consistently abolished these differences in DCs from different donors (Fig 1C and 1D). We conclude that a portion of the total DC population exhibits active, cytochalasin D-inhibitable, migration in a 3D collagen matrix over time. The 3D matrix set-up allows quantitative measurement of active DC migration over time.

### DCs challenged with *Toxoplasma* exhibit enhanced active migration in a 3D matrix

**Fig 2:**
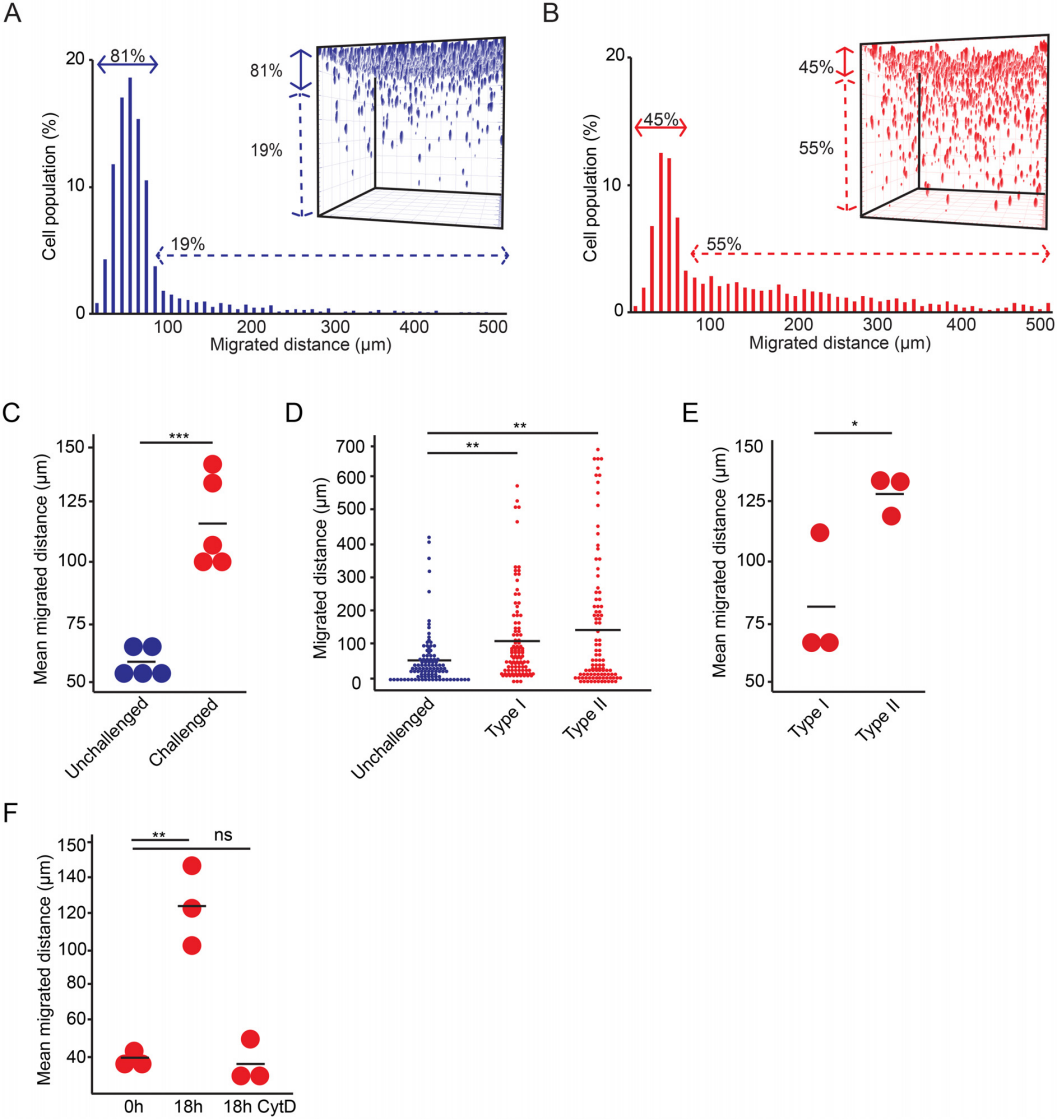
Migration in a 3D-matrix by DCs challenged with *T*. *gondii*. (**A** and **B**) Histograms represent the distribution of migrated distances for (**A**) unchallenged DCs in complete medium and (**B**) DCs pre-challenged with *T*. *gondii* (PRU-RFP, type II, MOI 3, 4h) as indicated under Materials and Methods. Arrows indicate, for each condition, the percentage (%) of cells in the matrix migrating < 80 μm or > 80 μm, respectively. 500 randomized cells from one representative donor are shown. The experiments were performed with DCs from 5 donors with similar results. Inset plots represent, for each condition, the 3D reconstruction assembly of z-stacks for the total cell population as indicated under Materials and Methods. (**C**) Mean migrated distances by unchallenged DCs and DCs challenged with *T*. *gondii* (PRU-RFP, type II), from 5 different donors. Data represent compiled analysis of 500 randomly chosen cells per donor. Bars indicate mean migrated distances. (***: P < 0.001; Paired t-test, Holm´s correction). (**D**) Dot plots represent the distribution of migrated distances of individual DCs challenged with *T*. *gondii* (type I: LDMluc; type II: PRU-RFP) related to unchallenged DCs. Bars indicate mean migrated distances. For each condition, 100 randomly chosen cells from one representative donor are shown. Significant differences were observed for challenged DCs (type I and II) versus unchallenged DCs (**: P < 0.01; Kruskal-Wallis test, Dunnett´s test). Performed with 3 donors with similar results. (**E)** Mean migrated distances of DCs challenged with *T*. *gondii* (type I: LDMluc; type II: PRU-RFP) as in (B) from 3 different donors. Data represent compiled analysis of 500 randomly chosen cells per donor. Bars indicate mean migrated distances. (*: P < 0.05; Paired t-test, Holm´s correction). (**F**) Mean migrated distances of DCs challenged with *T*. *gondii* (PRU-RFP, type II) as in (B) at indicated time points ± cytochalasin D (CytD). Data represent compiled analysis of 500 cells randomly chosen cells per donor from 3 different donors. (**: P < 0.01, ns: P > 0.05; Two-way ANOVA, Tukey´s HSD test).

We previously described that *Toxoplasma*-infected DCs exhibit a hypermotility phenotype and enhanced transmigration *in vitro* [7, 8]. However, *in vivo*, DC migration implies interactions with extracellular matrix in a 3D confinement. When migration of DCs was assessed in the 3D matrix setup, a higher portion of DCs challenged with *T*. *gondii* penetrated the matrix compared with unchallenged DCs (Fig 2A and 2B), with significant differences in mean migrated distances (Fig 2C). Representative strains of *T*. *gondii* type I and type II lineages induced enhanced migration of DCs compared to unchallenged DCs (Fig 2D) with significantly higher mean migrated distances for the type II strain (Fig 2E), at comparable infection frequencies (S2 Fig). Alike migration of unchallenged DCs (Fig 1), migration of DCs challenged with *T*. *gondii* was abolished by cytochalasin D-treatment (Fig 2F). We conclude that, in line with the previously described 2D hypermotility phenotype and enhanced transmigration in transwell systems, DCs challenged with *T*. *gondii* exhibit enhanced migration in the setting of a 3D collagen matrix.

### Differential morphology and migration of *Toxoplasma*-infected DCs and by-stander non-infected DCs

**Fig 3:**
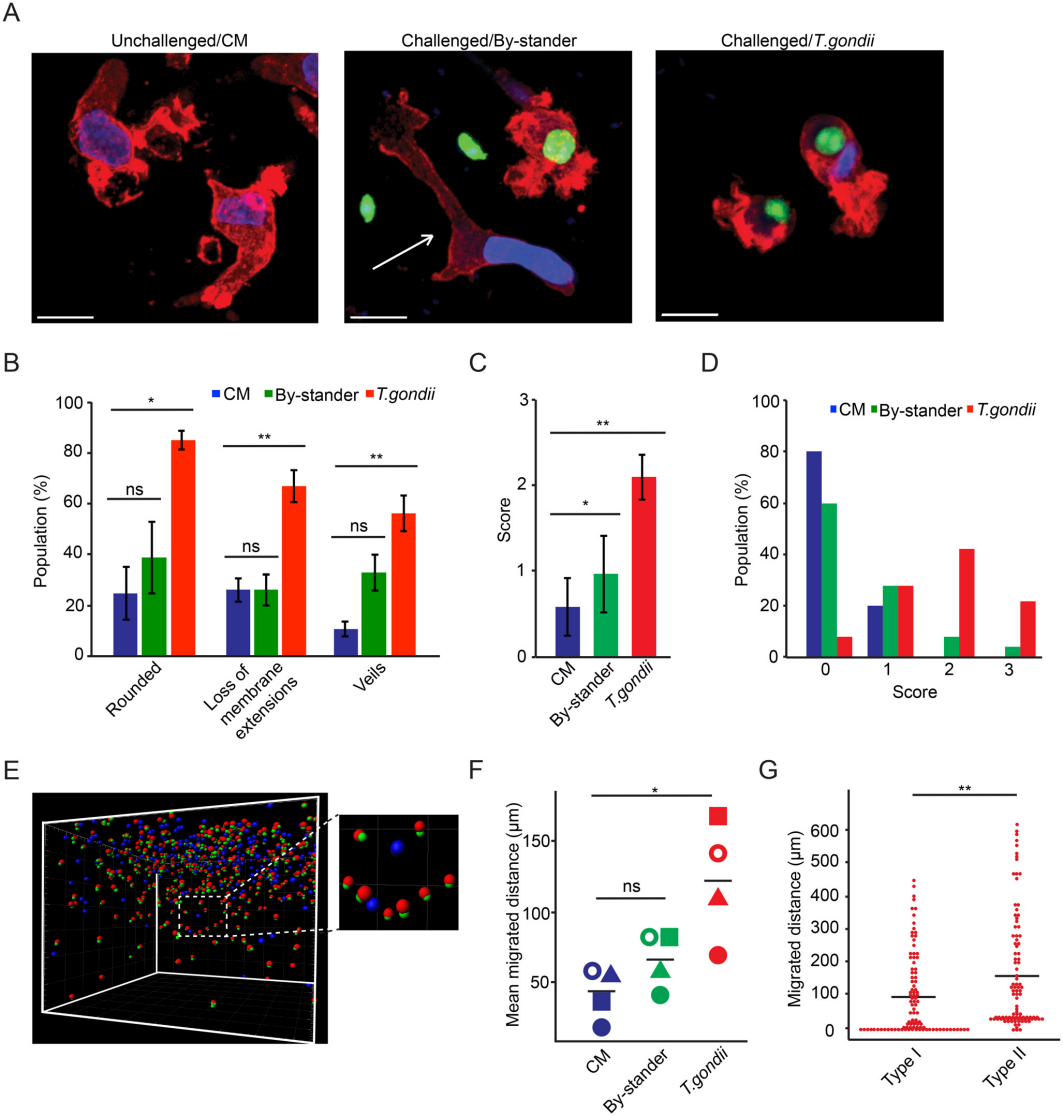
Morphological characteristics and migration of *Toxoplasma*-infected DCs and non-infected DCs in the 3D matrix. (**A**) Representative micrographs in maximum intensity projection of unchallenged DCs in complete medium (CM, left), Challenged/By-stander DCs (middle) and Challenged/*T*. *gondii* DCs (right; PTG-GFP **type II**, MOI 3, green) in a 3D collagen matrix, stained with DAPI (blue), and Alexa Fluor 594-Phalloidin (red) to detect F-actin as indicated under Materials and Methods. In the middle micrograph, arrow indicates non-infected DC surrounded by an infected DC (green + red) and two extracellular *T*. *gondii* tachyzoites (green). Scale bars = 10 μm. (**B**) Graph shows, for each condition, the percentage of cells (mean ±SEM) that exhibit rounded phenotype, absence of membrane extensions and veils, respectively, related to the total cell population. The morphological criteria are specified under Materials and Methods. For each condition, a total of 50 cells/donor were analyzed from 3 different donors. (*: P < 0.05, **: P < 0.01, ns: P > 0.5, Paired *t*-test, Holm´s correction). (**C**) Compiled mean scores (± SD) based on morphological criteria as in (B). For each condition, a total of 50 cells/donor were analyzed from 3 different donors (*: P < 0.05, **: P < 0.01, Paired *t*-test, Holm´s correction). (**D**) Distribution of the total scores (% of total cell population) based on morphological criteria specified under Materials and Methods. For each condition, a total of 50 cells/donor from 3 donors were assessed. Significant differences were observed between tachyzoite-infected DCs and non-infected DCs (P < 0.0001; Fisher´s exact test) or by-stander DCs (P < 0.0001), while differences between non-infected DCs and by-stander DCs were non-significant (P > 0.05). (**E**) Representative 3D projection analysis of DCs challenged with *T*. *gondii* (PTG-GFP type II). The colored spheres indicate the position of cells in the defined 3D space. Infected cells and non-infected cells were defined and analyzed as indicated under Materials and Methods: co-localized signal/infected cell (actin: red; *T*. *gondii*: green) or absence of co-localization/by-stander cells (blue). The inset image represents a magnification of the white-dotted square. Data are representative from 4 independent experiments. (**F**) Mean migrated distances by unchallenged DCs (CM), and challenged non-infected DCs (By-stander) and infected DCs (*T*. *gondii*: PTG-GFP type II). Data represent compiled analysis of 500 randomly chosen cells per donor from 4 different donors. Bars indicate mean migrated distances (*: P < 0.05, ns: P > 0.05, Two-way ANOVA, Tukey´s HSD test). (**G**) Dot plots represent the distribution of migrated distances for individual DCs infected with *T*. *gondii* (type I: LDMluc; type II: PRU-RFP). For each condition, 100 single cells were randomly selected and analyzed from one representative donor. Bar indicates mean migrated distance. Asterisks indicate significant differences (**: P < 0.01; Paired *t*-test, Holm´s correction).

Because DCs display different contractile and protrusive forces during 3D migration versus 2D migration [18], we analyzed the features of infected DCs and non-infected DCs in the matrix using morphological criteria –cell shape, dendrite-like extensions and membrane veils/ruffling [4]. Consistently, unchallenged DCs and challenged by-stander (non-infected) DCs exhibited elongated shape and membrane extensions (Fig 3A), while infected DCs exhibited a rounded shape with veils/ruffles and absence of membrane extensions (Fig 3A). Importantly, infected DCs presented significantly elevated morphology scores and could be easily distinguished from unchallenged DCs and by-stander DCs, while by-stander DCs resembled unchallenged DCs in the matrix (Fig 3B, 3C and 3D). Next, we analyzed the migrated distances by infected DCs and by-stander DCs (Fig 3E, S1 Video). In separate donors, significantly higher mean migrated distances were observed in infected DCs compared to unchallenged DCs and by-stander DCs (Fig 3F). Corroborating earlier observations (Fig 2D and 2E), DCs infected by type II parasites migrated significantly longer distances compared to DCs infected by type I parasites (Fig 3G). We conclude that, in the 3D matrix, *Toxoplasma*-infected DCs exhibit morphological features consistent with rounded/amoeboid morphology and enhanced penetration of the matrix compared to by-stander DCs. The amoeboid features, including enhanced migration, are significantly more pronounced in infected DCs related to non-infected by-stander DCs.

### *Toxoplasma*-infected DCs exhibit integrin-dependent adhesion but migrate in the 3D matrix in an integrin-independent fashion

**Fig 4:**
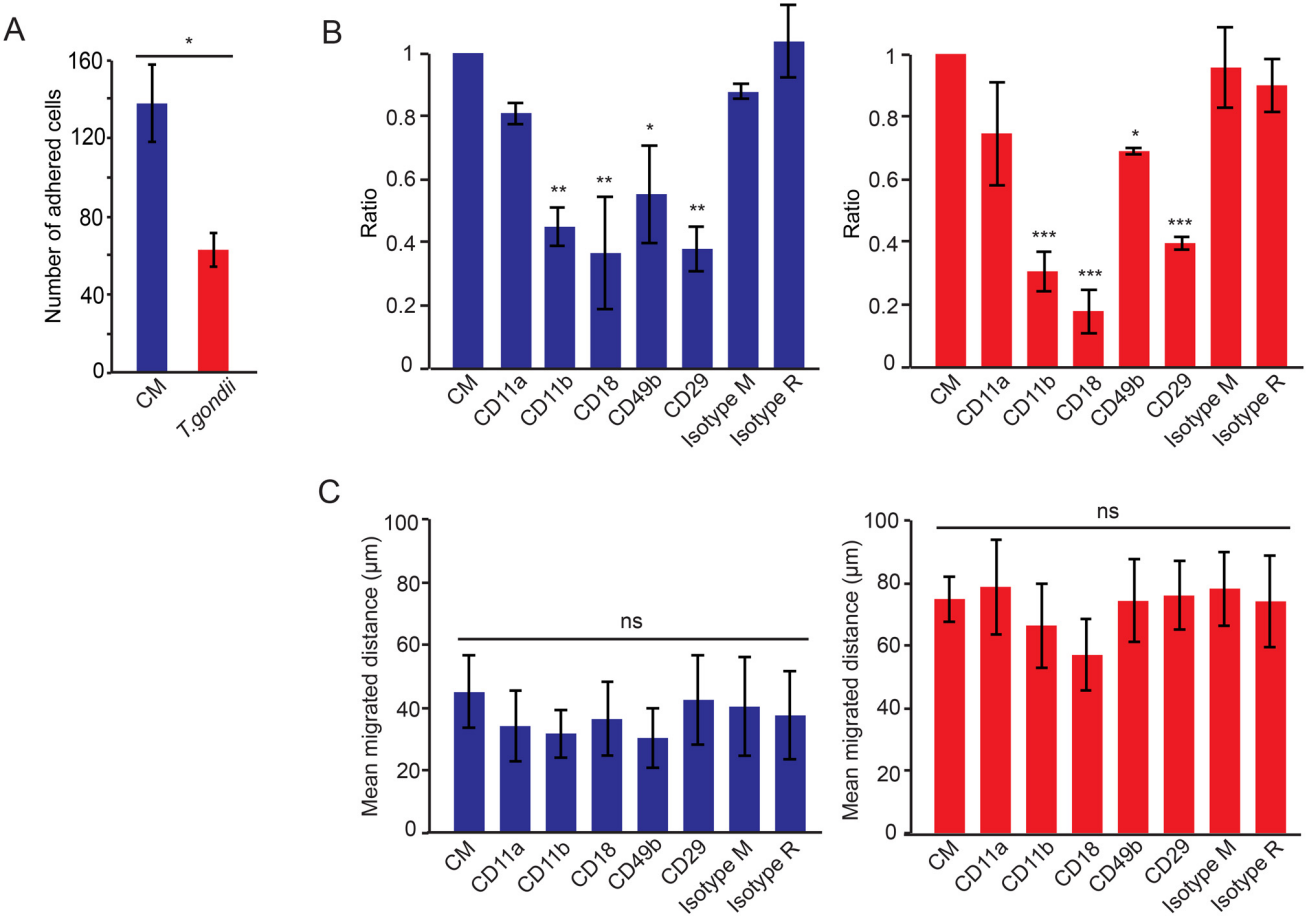
Adhesion and migration of *Toxoplasma*-infected DCs in the presence of integrin-blocking antibodies. (**A**) Bar graph shows average number of adhered cells per 100 mm^2^ (± SD) for unchallenged DCs (CM) and *Toxoplasma*-challenged DCs (*T*. *gondii*, PRU-RFP **type II**, MOI 3, 4h) as indicated under Materials and Methods (*: P < 0.05; Paired *t-*test, Holm´s correction).(**B**) Bar graphs show the ratio of adhered cells treated with blocking antibodies compared to cells in CM from 3 donors. Unchallenged DCs (left/blue) and DCs challenged with *T*. *gondii* (right/red, PRU-RFP, MOI 3, 4h) were exposed to anti-human CD11a, anti-human CD11b, anti-human CD18, anti-human CD29, anti-human CD49b, as indicated under Materials and Methods, for 30 min and seeded on 1% BSA/serum-coated plates (CD11a, CD11b, CD18) or collagen-coated plates (CD29, CD49b). Mouse IgG1 κ Isotype (Isotype M, 10 μg/ml) or Rat IgG2b κ Isotype (Isotype R, 10μg/ml) were used as control antibodies (*: P < 0.05, **: P < 0.01, ***: P < 0.001,Two-way ANOVA, Dunnett´s test). (**C**) Mean migrated distances in 3D collagen matrix by cells exposed to blocking antibodies as in (B). Graphs show unchallenged DCs (left/blue) and DCs challenged with *T*. *gondii* (right/red, PRU-RFP, MOI 3, 4h). Data represent compiled analysis of 400 cells/donor (± SEM) from 4 donors. (ns: P > 0.05; Two-way ANOVA).

As DCs utilize both integrin-dependent and integrin-independent migration modes [18], we sought to determine the implication of integrins in the observed enhanced migration of *Toxoplasma*-infected DCs in collagen. Also, the interaction of cells with extracellular matrix collagen may be direct (collagen-integrin interaction) or indirect (collagen-serum proteins-integrin) [24]. We therefore assessed the impact of integrins known to bind to collagen (CD29, CD49b) and serum proteins (CD11a, CD11b, CD18) in a static adhesion assay, using blocking antibodies [23]. In absence of blocking antibodies, infected DCs exhibited significantly reduced adhesion compared with non-infected DCs (Fig 4A), which is in line with previous morphological analyses in 2D confinements [4]. Next, adhesion of infected and non-infected DCs was assessed in the presence of integrin-blocking antibodies (CD11a, CD11b, CD18, CD29, CD49b). In this assay, blocking antibodies to the serum protein-binding integrin subunits CD11b, CD18 and the collagen-binding integrin subunits CD29, CD49b had a profound inhibitory effect on adhesion of both infected and non-infected DCs (Fig 4B). In sharp contrast, in the 3D collagen matrix, an absence of inhibitory effect on migration was observed for both infected and non-infected DCs (Fig 4C). We conclude that integrin-blocking antibodies do not significantly inhibit the migration of infected and non-infected DCs in the 3D matrix. The results are in line with the described features of DCs during integrin-independent amoeboid migration [18].

### CCL19 potentiates the hypermigratory phenotype of *Toxoplasma*-infected DCs in a 3D matrix

**Fig 5:**
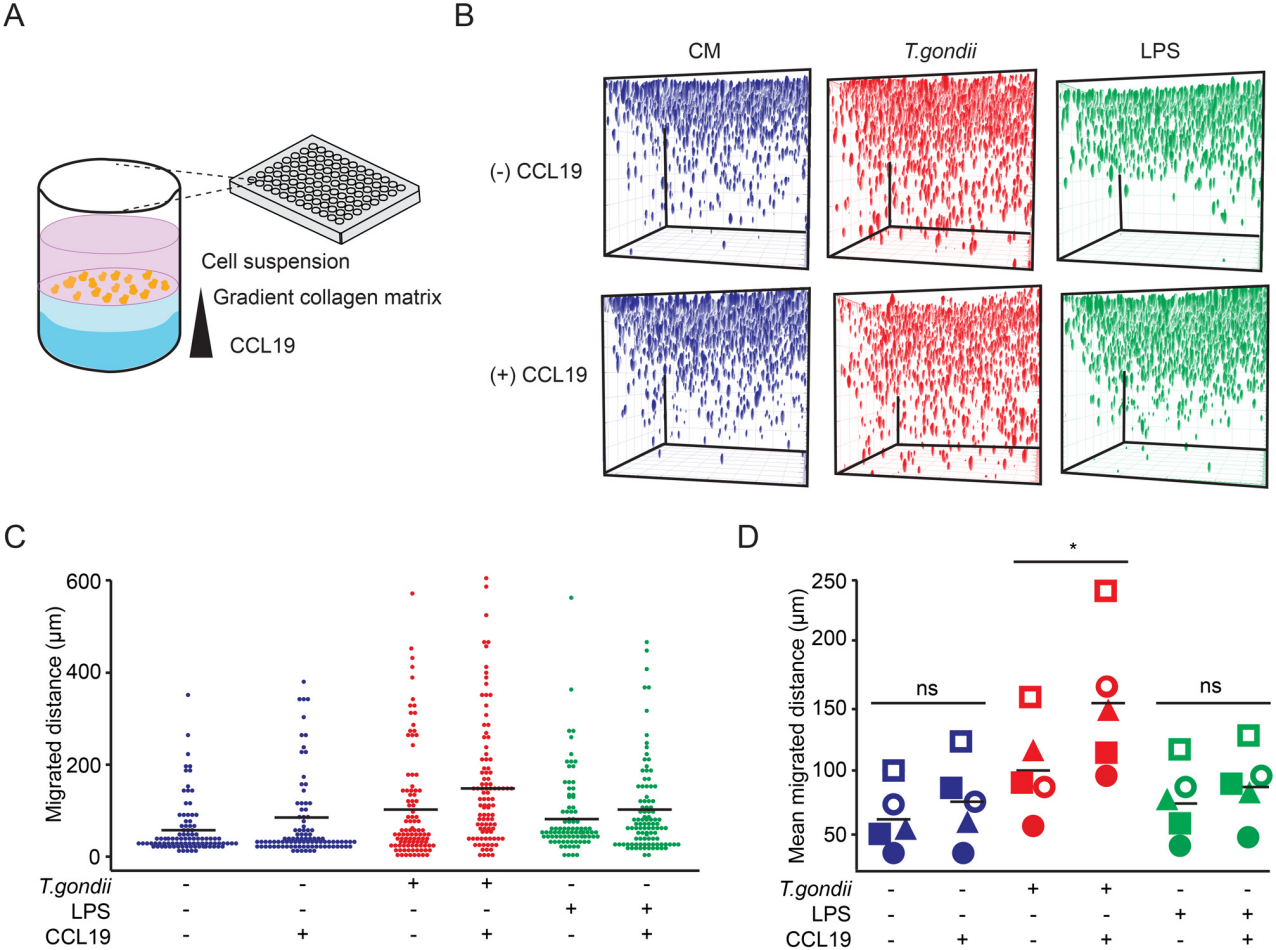
Chemotaxis and hypermigration of *Toxoplasma*-infected DCs in a 3D matrix. (**A**) Schematic representation of the assay set-up with CCL19 added in the lower collagen matrix (dark blue). Cells were deposited onto the upper collagen matrix (light blue) as indicated under Materials and Methods. DCs were maintained in CM, pre-challenged with *T*. *gondii* (PRU-RFP, type II) or treated with LPS (final concentration 100 ng/ml) and deposited on top of the collagen layer in 96-well plates. After 24 h incubation, the localization of DAPI-labeled DCs in the gel was analyzed in 200 z-sections. (**B**) Plots indicate the assembly of z-stacks and colored structures indicate the localization of DCs in absence or presence of CCL19. (**C**) Dot plots represent the distribution of migrated distances for the different conditions. For each condition, 100 randomly chosen cells were analyzed from one representative donor. Performed with 5 donors with similar result. (**D**) Mean migrated distances of cells under same conditions as in B and C. Data represent compiled analysis of 500 randomly chosen cells per donor from 5 different donors. Bars indicate mean migrated distances. (*: P < 0.05, ns: P > 0.05; Paired *t*-test, Holm´s collection).

We have previously shown that the hypermigratory phenotype of infected DCs does not depend on chemotactic cues, e.g. CCR7 or CCR5 [5] but that *Toxoplasma*-infected DCs can (independently of hypermotility) up-regulate CCR7, down-regulate CCR5 and chemotax in a 2D setting [4]. While these studies showed that hypermotile *Toxoplasma*-infected DCs can be directionally guided by a chemokine gradient [4, 7], they did not provide a quantitative measurement as to whether hypermotility was potentiated by CCL19, i.e. whether the migrated distances were further enhanced in the presence of chemokine. To address this, a chemokine gradient was generated in the 3D matrix by a sandwich procedure (Fig 5A) and infected DCs and LPS-treated DCs were allowed to migrate in this gradient. In the presence of the CCR7-ligand CCL19, a relative increase of migrated distances towards the chemokine gradient was observed for all conditions (Fig 5B and 5C). In 5 independent human donors tested, DCs challenged with *T*. *gondii* exhibited significant elevations of mean migrated distances (Fig 5D). For LPS-treated DCs, a non-significant elevation of mean migrated distances was consistently observed in presence of CCL19 for the total cell population (Fig 5C) and significantly elevated mean migrated distances were observed when comparing highly migratory cells (defined as > 100 μm penetration of the matrix; S3 Fig). We conclude that, while this modality of the assay was not designed to quantitatively discriminate between chemotactic and chemokinetic effects, *Toxoplasma*-induced enhanced migration of DCs can be further potentiated by CCL19 in a 3D matrix.

## Discussion

Previous work has established that *Toxoplasma*-infected DCs contribute to parasite dissemination in mice and that infected DCs exhibit hypermotility in a 2D confinement and enhanced transmigration in transwell systems *in vitro*. However, the shuttling function of infected DCs *in vivo* implies passage across endothelial cellular barriers and interstitial migration in extracellular matrix. While the passage of infected DCs across endothelial cell monolayers has been partly addressed *in vitro* [5], the behavior of *Toxoplasma*-infected DCs in extracellular matrix remained unexplored. Here, we have analyzed the migration of *Toxoplasma*-infected DCs in a collagen matrix and report that a hypermigratory phenotype is induced in infected DCs in a 3D confinement.

The established assay offers a relatively simple 96-well plate setup with high reproducibility between experiments or donors and is combined with a semi-automated analysis algorithm. One of the advantages of 3D matrix systems is that they mimic the *in vivo* conditions where cells are surrounded by extracellular matrix. Although collagen is the major protein component of the extracellular matrix, its native composition is far more complex. However, for reproducibility and reduction of variability in the assay, we opted to use formulated collagen and cell culture medium supplemented with FBS, which contains many of the components present in extracellular matrix. The assay also circumvents or complements some of the limitations of other assays. Transmigration assays measure cellular transmigration over time across pores, of variable size and density, in synthetic filter membranes, i.e., the locomotion of cells that fail finding a pore and consequently do not transmigrate is not taken into account [5]. Motility assays on coated plastic or glass surfaces take into consideration locomotion of the total cell population but 2D confinements likely over-emphasize adhesion-dependent motility processes [4, 18, 20]. Advantages with the 3D matrix assay may be that it allows both measurement of migration of individual cells and analysis of the total cell population over time in a 3D confinement that resembles the interstitial environment. While technically relatively uncomplicated, a disadvantage may be that the assay requires advanced imaging equipment and analysis software. A limitation of this assay is that it provides the Euclidian distance between the start and end points, thereby probably slightly underestimating the total migrated distances of individual cells. The modality of the assay with a chemokine gradient has the limitations that it measures directionality only towards the chemokine gradient, and endpoint migrated distances. Thus, in the strict sense, this modality does not discriminate between chemotaxis and chemokinesis. Overall, for high throughput comparisons of cell populations, the assay exhibited high reproducibility within sets of experiments, acceptable reproducibility between sets of experiments and acceptable variability between different human donors.

It has been shown that, in order to perform rapid migration in matrix, DCs utilize an integrin-independent amoeboid type of migration [18]. We previously reported that integrin expression levels in *Toxoplasma*-infected DCs are down modulated or maintained [5], and that redistribution of integrins to the edges of infected DCs occurs in 2D confinements [4]. Here, we show that infected DCs exhibit significantly lower adhesion frequency to collagen- and BSA-coated surfaces compared to non-infected DCs but that the adhesion can be competed out by integrin blockade. This is indicative that integrin-mediated adhesion of infected DCs may be important for binding to endothelial cells upon transmigration, as shown in monocytic cells [25]. In contrast, integrin-blocking antibodies had non-significant effects on 3D migration of DCs under conditions that significantly blocked adhesion to coated 2D surfaces. This, together with the observed morphological changes, is strongly indicative of high-speed integrin-independent amoeboid type of migration [18]. Also, integrin-blocking antibodies insignificantly inhibited the migration of non-infected DCs, suggesting that the migratory fraction of non-infected DCs in the collagen matrix also may use amoeboid migration in response to environmental cues [18, 26] and that *Toxoplasma* infection exacerbates this migratory phenotype. We consider that the accessibility of integrin blocking-antibodies to cells in the matrix is good, given the good accessibility of phalloidin-RFP/GFP when performing labeling for immunofluorescence and earlier reports using antibodies [27, 28].

Morphological analyses of DCs migrating in the matrix provided additional key elements. We previously reported that shortly after parasite invasion, dramatic morphological changes take place in infected DCs in a 2D setup, with rounding up, loss of podosomes and appearance of membrane veils as prominent signs [4]. Because podosomes limit fast migration by their strong interactions with the extracellular matrix, the presence of podosomes appears incompatible with the high-speed amoeboid migration observed in mature DCs [29–31]. This amoeboid motility of DC occurs independently of adhesion to specific substrates and extracellular matrix degradation [18] and is required for efficient migration [19]. We previously reported that podosomes rapidly dissolve in DCs upon infection by *T*. *gondii* [4]. In line with observations of DCs in 3D set-ups [18] but in contrast to observations in 2D assays [4], podosomes were absent in uninfected (and in infected) DCs migrating in the 3D matrix. This is in agreement with the absence of inhibition by integrin-blockade for both infected and uninfected DCs. Additionally, infected DCs acquired a rounded morphology with prominent veils and ruffles, all in line with observations in 2D confinements [4]. Thus, the lack of dependency on integrin-binding together with the distinct morphological features, strongly advocate for the onset of an amoeboid-like type of migration by *Toxoplasma*-infected DCs.

*Toxoplasma*-infected DCs also exhibited significantly enhanced penetration of the matrix compared to non-infected by-stander DCs, which more resembled unchallenged DCs in respect of morphological features and migrated distances. This corroborates the observed lack of, or minimal, by-stander effect in 2D analyses of DC hypermotility and transmigration in transwell inserts [4, 5, 7]. It also indicates that the induced hypermigratory phenotype in the infected DC is tightly regulated by the infecting parasite. Here, we extend these findings and show that two type II strains induces a more pronounced hypermigratory phenotype compared to a type I strain in the 3D matrix, in line with previous observations in transmigration systems [8].

We have previously shown that *Toxoplasma*-induced hypermotility of DCs sets in within minutes after infection and that the hypermigratory phenotype does not depend on classical chemotactic activation, e.g. in LPS-matured DCs [4, 5, 7]. Interestingly, and not in contraposition, infection of DCs by *T*. *gondii* leads to an up-regulation of CCR7 and down-regulation of CCR5 [4, 7], and also up-regulation of co-stimulatory molecules and cell surface maturation markers [5]. Altogether, this indicates that infection *per se* also leads to maturation events that make the DCs responsive to chemotactic cues. Importantly, up-regulation of CCR7 was not observed in by-stander DC indicating a direct effect of intracellular parasite localization and that activation required prolonged exposure [4, 7]. Thus, *Toxoplasma*-induced hypermotility of DCs and chemokine-induced migration of infected DCs are two mechanisms that can be distinctly separated in time *in vitro* [3, 4, 7]. Chemokines, e.g. CCL19 acting on CCR7, induce both chemotactic and chemokinetic stimuli in immune cells [32–34]. Despite that our assay cannot with certainty discriminate between chemotactic and chemokinetic effects, a significant increase in migrated distances was consistently observed in presence of CCL19, suggesting that hypermotile *Toxoplasma*-infected DCs respond to this chemokine with enhanced migration in a 3D matrix and in line with observations in 2D settings [4, 7]. Thus, we show that *Toxoplasma*-induced hypermigration of DCs and CCR7-mediated stimuli can work in conjunction in a 3D matrix, thereby potentiating the migration of infected DCs further. These results may have implications for how we conceive dissemination of *T*. *gondii*. Hypothetically, *Toxoplasma*-induced amoeboid-like migration and CCR7-mediated chemotaxis and chemokinesis could cooperatively enhance the migratory potential of infected DCs *in vivo*, and consequently, potentiate the dissemination of the parasite. This is well in line with the observed enhanced dissemination upon adoptive transfers of infected DCs [5, 8, 35] and the inhibitory effects on DC migration by pertussis toxin treatment [5, 36]. However, the study of the mechanistic interplay between hypermotility and chemotaxis awaits further investigation.

In summary, our study provides a novel assay for *in vitro* quantification of cell migration in the context of Toxoplasma infection and reports that amoeboid-like migration is induced in infected DCs. The assay may be of value for better understanding how invasive microorganisms breach biological barriers and how intracellular pathogens manipulate immune cells for dissemination. Understanding how *Toxoplasma* manipulates the modes of locomotion of leukocytes may provide new insights into the mechanisms of dissemination and the pathogenesis of disease.

## Supporting Information

S1 Fig Automated determination of single DC position in the 3D matrix. DCs (5×10^4^) were applied to the top of the collagen matrix and incubated for 18 h. After fixation and DAPI-staining, image projections were created from the imaged 3D space (x, y, z planes were 848.53 μm, 848.53 μm and 692.52 μm respectively) and the position of individual DCs in the matrix 3D confinement was determined as indicated under Materials and Methods. **(A)** Orthogonal maximum intensity projection (z plane) and magnified inset micrograh depict the cell density of DAPI-stained cells (blue) detected by the Imaris spot function (red). **(B)** Sagital maximum intensity projection of (A) shows higher cell density in the upper part of the matrix related to the lower part. Inset illustrates, as in (A), detection of cells that have penetrated the matrix.
(TIF)

S2 Fig Determination of DC infection frequencies with type I and type II strains. DCs were challenged with freshly egressed tachyzoites for 4h at MOI 3 as indicated under Materials and Methods. Cells suspensions were seeded on poly-L-lysine-coated coverslips, fixated and stained with fluorochrome-labeled phalloidin. Micrographs of random fields of view were taken by epifluorescence microscopy and infection frequency was visually determined by counting 150–200 cells. **(A)** Micrograph depicts DCs stained with phalloidin-RFP (red) and DAPI (blue) challenged with GFP-expressing LDMluc (type I, green). Scale bar 20 μm. Mean infection frequency (% ± SD) was 51% ± 8. **(B)** Micrograph depicts DCs stained with phalloidin-GFP (green) and DAPI (blue) challenged with RFP-expressing PRU (type II, red). Scale bar 20 μm. Mean infection frequency (% ± SD) was 50% ±6.
(TIF)

S3 Fig Migration of LPS-treated DCs in presence of CCL19. DCs were deposited on top of the sandwich collagen matrix containing CCL19 as indicated under Materials and Methods and Fig 5A. After 24 h incubation in the presence of LPS (100 ng/ml), the localization of DAPI-labeled DCs in the gel was analyzed in 200 z-sections as indicated. (**A**) Histograms represent the distribution of migrated distances for LPS-treated DCs in absence (-) or presence (+) of CCL19, respectively. For each condition, 500 randomized cells migrating > 100 μm from one representative donor are shown. (B) Mean migrated distances of cells under same conditions as in (A). Data represent compiled analysis of 500 randomly chosen cells per donor from 3 different donors. Bars indicate mean migrated distances. (*: P < 0.05; Paired *t*-test, Holm´s correction).
(TIF)

S1 Video 3D projection analyses of DCs challenged with *T*. *gondii* in a collagen matrix. Infected and non-infected cells were defined and analyzed as indicated under Materials and Methods. The colored spheres indicate co-localized signal (actin: red; *T*. *gondii* type II: green) or absence of co-localization/by-stander non-infected cells (blue). Representative data from 5 independent experiments are shown.
(MOV)
