# Supplementary figures and images for: Infection by Toxoplasma gondii Induces Amoeboid-Like Migration of Dendritic Cells in a Three-Dimensional Collagen Matrix
Source: PLoS One. 2015 Sep 25;10(9):e0139104. doi: 10.1371/journal.pone.0139104 (PMC4583262; doi:10.1371/journal.pone.0139104)

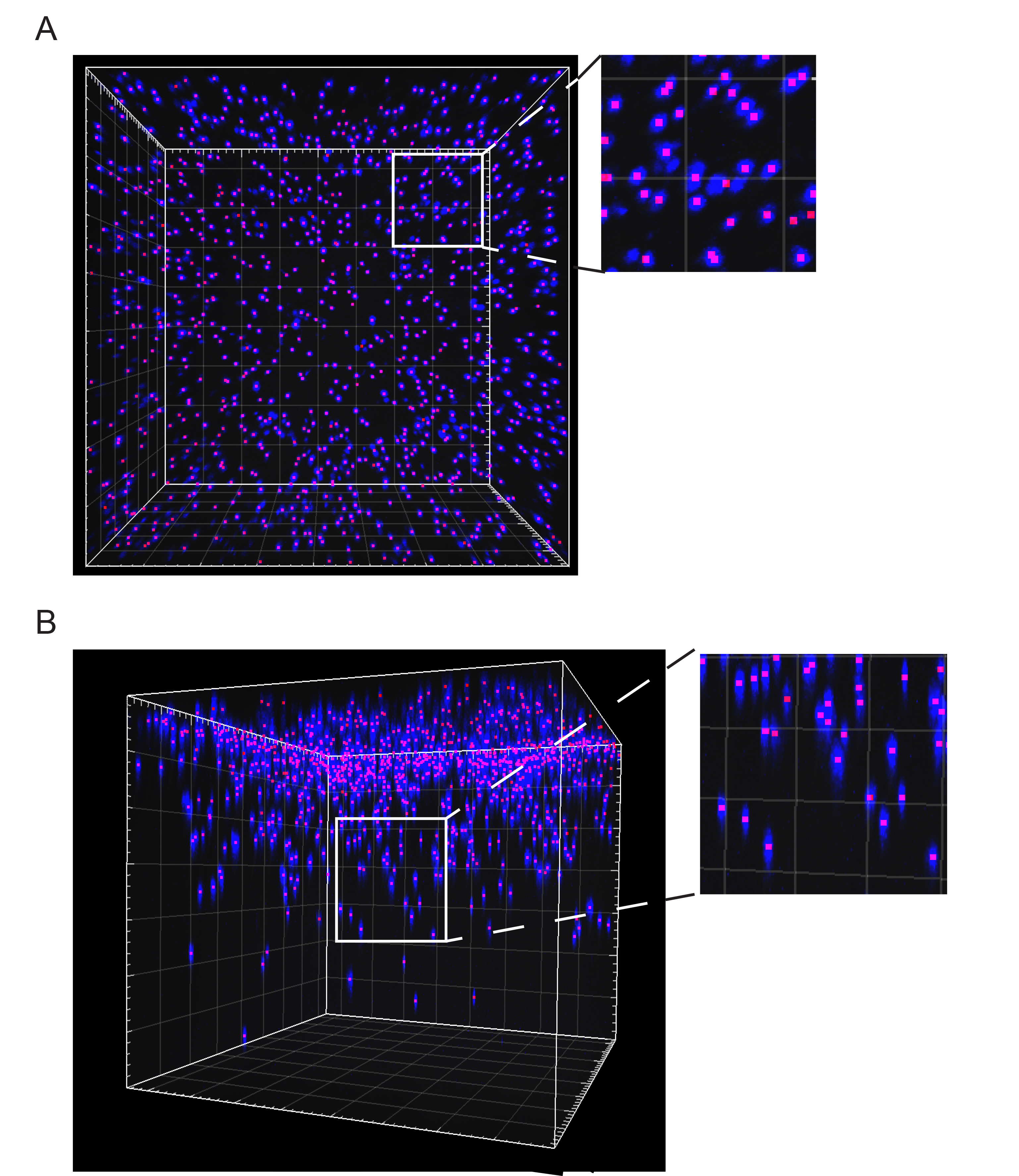

Supplement: S1 Fig — DCs (5×10^4^) were applied to the top of the collagen matrix and incubated for 18 h. After fixation and DAPI-staining, image projections were created from the imaged 3D space (x, y, z planes were 848.53 μm, 848.53 μm and 692.52 μm respectively) and the position of individual DCs in the matrix 3D confinement was determined as indicated under Materials and Methods. (A) Orthogonal maximum intensity projection (z plane) and magnified inset micrograh depict the cell density of DAPI-stained cells (blue) detected by the Imaris spot function (red). (B) Sagital maximum intensity projection of (A) shows higher cell density in the upper part of the matrix related to the lower part. Inset illustrates, as in (A), detection of cells that have penetrated the matrix. (TIF) [file pone.0139104.s001.tif]

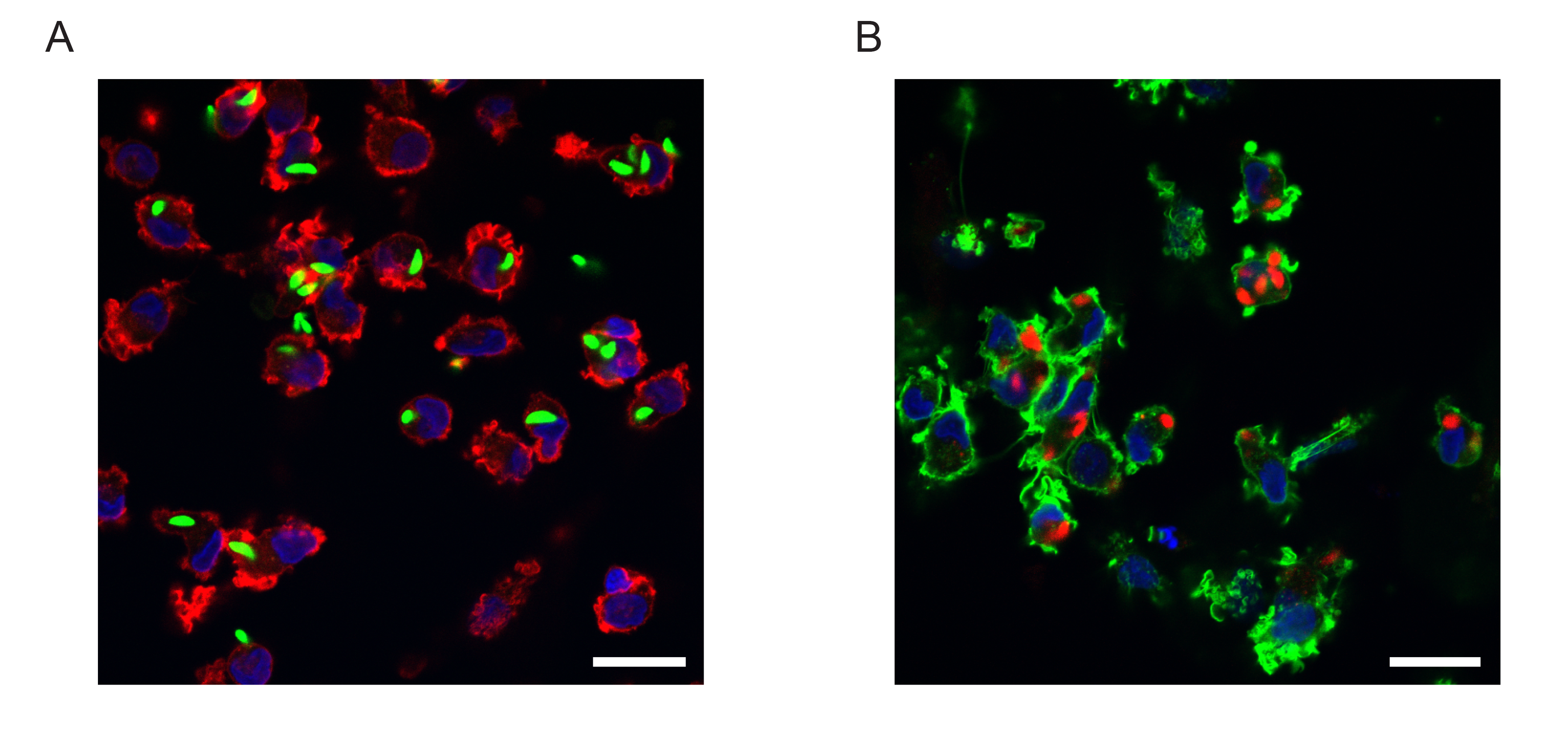

Supplement: S2 Fig — DCs were challenged with freshly egressed tachyzoites for 4h at MOI 3 as indicated under Materials and Methods. Cells suspensions were seeded on poly-L-lysine-coated coverslips, fixated and stained with fluorochrome-labeled phalloidin. Micrographs of random fields of view were taken by epifluorescence microscopy and infection frequency was visually determined by counting 150–200 cells. (A) Micrograph depicts DCs stained with phalloidin-RFP (red) and DAPI (blue) challenged with GFP-expressing LDMluc (type I, green). Scale bar 20 μm. Mean infection frequency (% ± SD) was 51% ± 8. (B) Micrograph depicts DCs stained with phalloidin-GFP (green) and DAPI (blue) challenged with RFP-expressing PRU (type II, red). Scale bar 20 μm. Mean infection frequency (% ± SD) was 50% ±6. (TIF) [file pone.0139104.s002.tif]

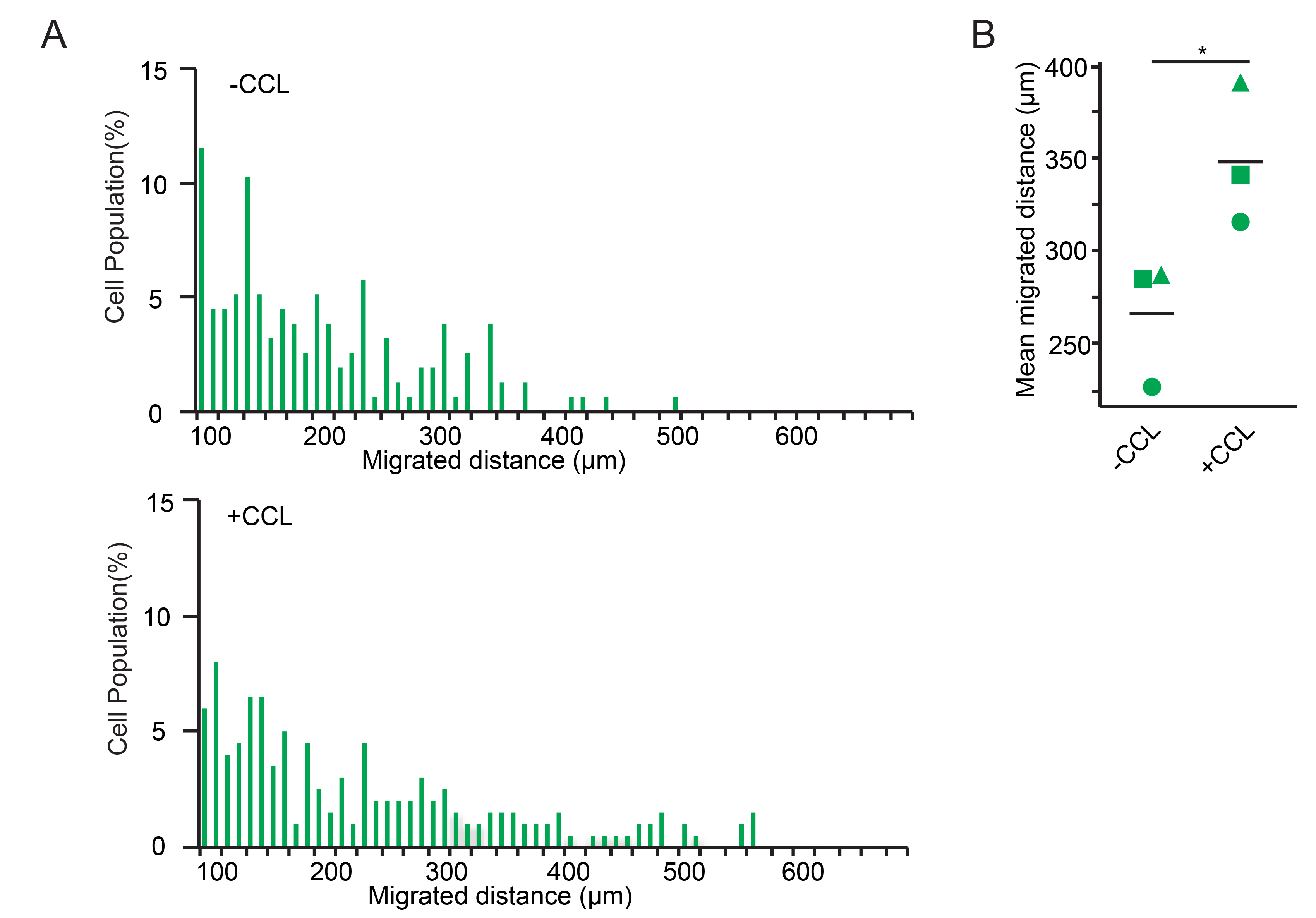

Supplement: S3 Fig — DCs were deposited on top of the sandwich collagen matrix containing CCL19 as indicated under Materials and Methods and Fig 5A. After 24 h incubation in the presence of LPS (100 ng/ml), the localization of DAPI-labeled DCs in the gel was analyzed in 200 z-sections as indicated. (A) Histograms represent the distribution of migrated distances for LPS-treated DCs in absence (-) or presence (+) of CCL19, respectively. For each condition, 500 randomized cells migrating > 100 μm from one representative donor are shown. (B) Mean migrated distances of cells under same conditions as in (A). Data represent compiled analysis of 500 randomly chosen cells per donor from 3 different donors. Bars indicate mean migrated distances. (*: P < 0.05; Paired t-test, Holm´s correction). (TIF) [file pone.0139104.s003.tif]
